# Supplementary material for: Global radiation in a rare biosphere soil diatom
Source: Nat Commun. 2020 May 13;11:2382. doi: 10.1038/s41467-020-16181-0 (PMC7221085; doi:10.1038/s41467-020-16181-0)
Supplement: Supplementary file 1 — Supplementary Information [file 41467_2020_16181_MOESM1_ESM.pdf]

Supplementary Information

GLOBAL RADIATION IN A RARE BIOSPHERE SOIL DIATOM

PINSEEL ET AL.

This Supplementary Information file includes the following items:

- Supplementary Figures 1 – 13
- Supplementary Tables 1 – 3
- Supplementary References

Supplementary Figures

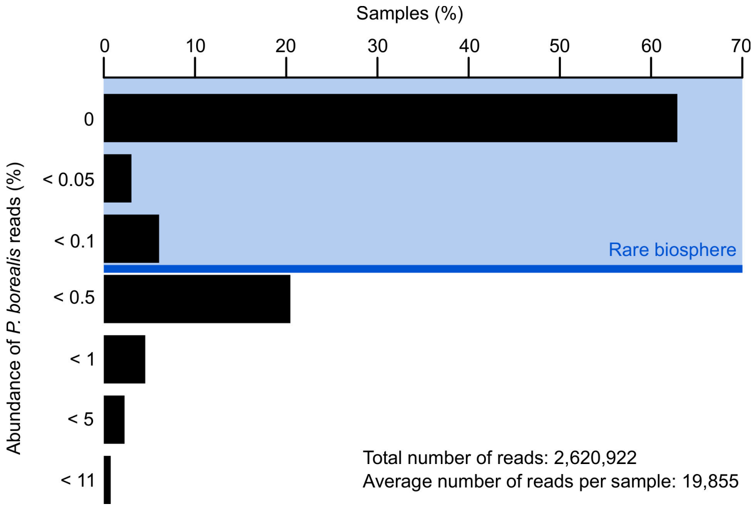

Supplementary Figure 1 | Environmental metabarcoding of *P. borealis*. The barplots show the percentage of environmental samples with a given abundance of *P. borealis* 18S-reads within the whole protist community. In all samples, the presence of *P. borealis* (living cells or cells with distinct cell content) was confirmed by observation in light microscopy or detection in a previous metabarcoding study. Following ref.[1] the threshold of the rare biosphere is set at a maximum abundance of 0.1%, indicating that in the majority of the samples containing *P. borealis*, the complex belongs to the rare biosphere. For these calculations, all reads belonging to the Embryophyceae were removed prior to the analysis. Source data are provided as a Source Data file.

a

	28S	*cox* 1	Extra information
Number of strains	850	584	
SPNA	88	127	95% connection limit; gaps treated as missing data
ABGD	78	130	28S: first recursive partition; *cox* 1: initial partition
sGMYC	89 (86–95)§	127 (110–131)	28S: L_0* = 2164.4, L_{GMYC}† = 2191.2, LR = 53.7, LR‡ test p-value = 2.233e-12; *cox* 1: L_0 = 1355.2, L_{GMYC} = 1467.6, LR = 224.9, LR test p-value = 0
PTP	95	128	500,000 generations; 25% burnin
bPTP	98	128	500,000 generations; 25% burnin
Singletons‖	50	73	Many haplotypes are represented by multiple individuals
Consensus¶	84	126	Delimited by at least four out of five methods

* Likelihood null model
† Maximum likelihood of the sGMYC model
‡ Likelihood ratio
§ 95% confidence interval
|| Number of delineated consensus lineages that are represented by a single haplotype
¶ Number of consensus lineages, delimited by at least four out of five methods

b **c**

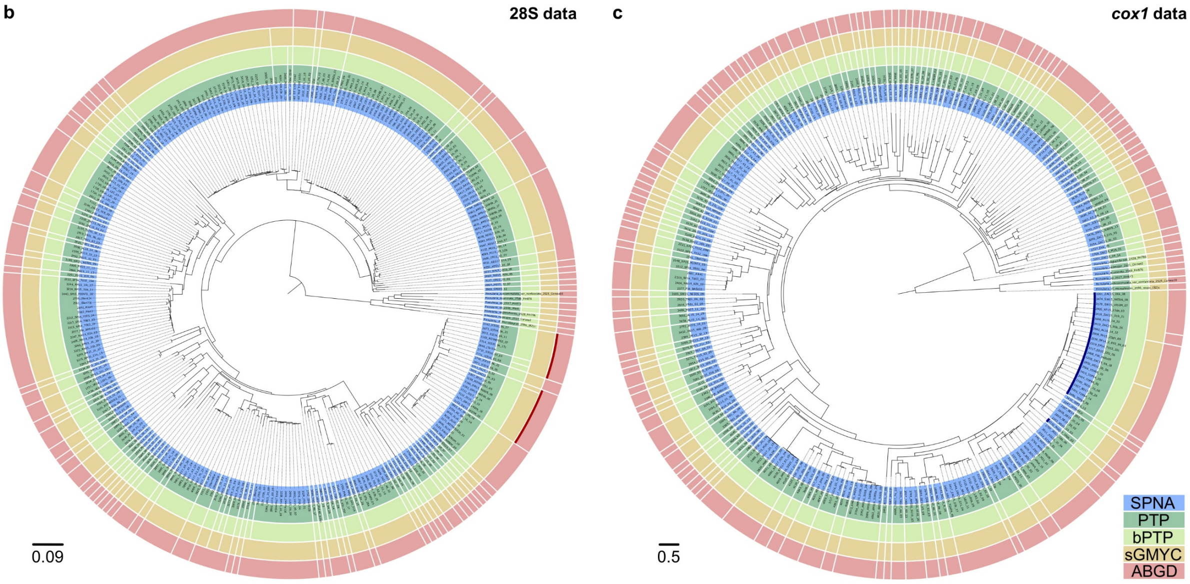

Supplementary Figure 2 | Results of the single-locus automated species delimitation analyses. a, Table showing the number of putative species delimited by each method, for both loci separately. **b**, Figure showing the results of the species delimitation on the 28S-dataset. **c**, Figure showing the results of the species delimitation on the *cox1*-dataset. In **b** and **c**, all delimited putative species are depicted for each method separately. Putative species that were paraphyletic for a given method are indicated by dark-colored bars (red in **b** and blue in **c**). The depicted ML phylogenies were calculated in RAxML and include one strain per unique haplotype. Five methods for species delimitation were used: Statistical Parsimony Network Analysis (SPNA), (Bayesian) Poisson Tree Processes ((b)PTP), General Mixed Yule Coalescent (sGMYC), and Automated Barcode Gap Discovery (ABGD).

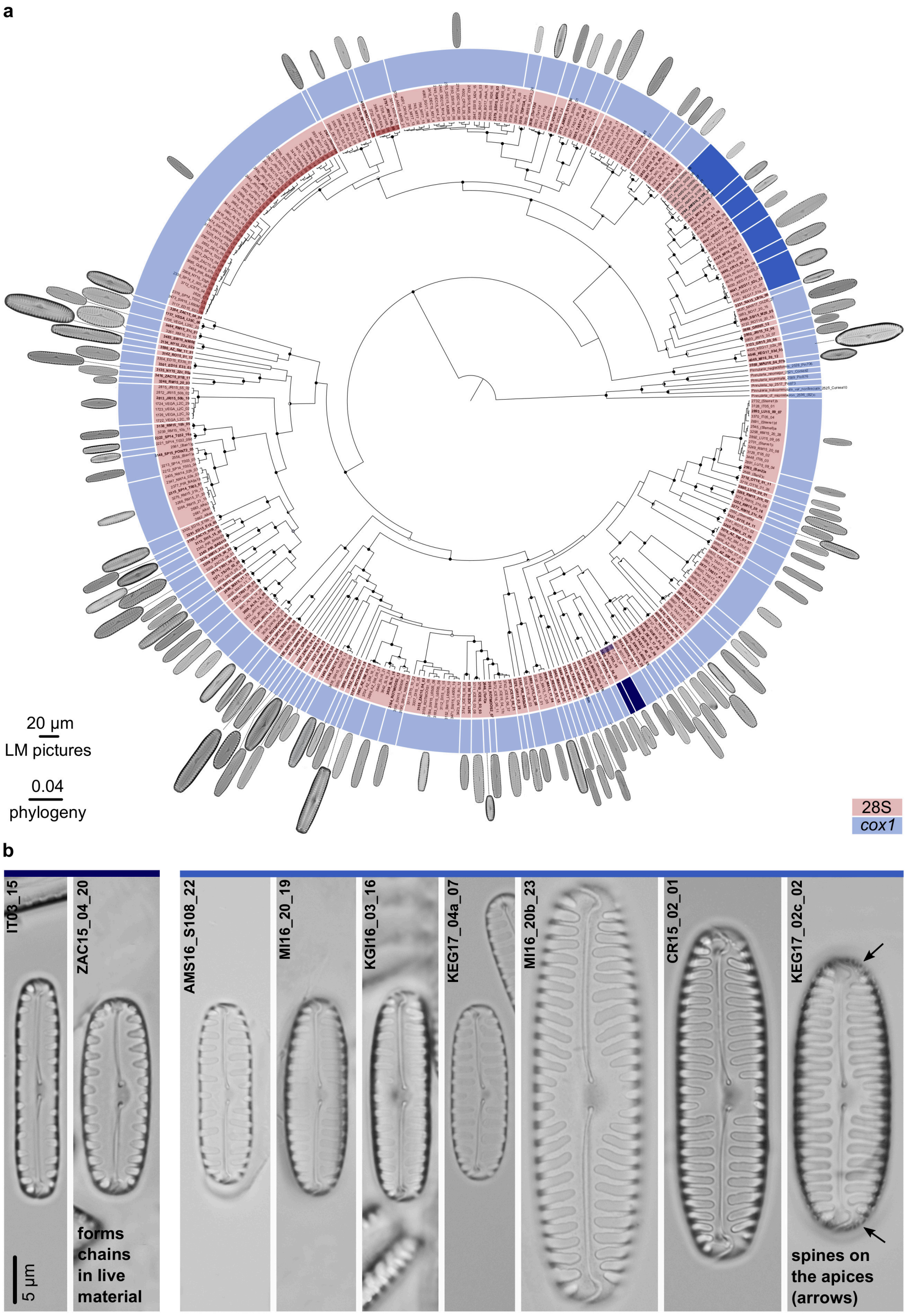

Supplementary Figure 3 | Consensus species delimitation. a, Bayesian phylogeny (BEAST) of the concatenated haplotype dataset of 28S and *cox1*, with indication of the lineages delimited by each gene. Two 28S-lineages are paraphyletic in the BEAST phylogeny (thin red and blue lines). For each lineage, one light microscopy (LM) picture of the representative strain (bold) is given, showing a cell wall in perpendicular view after removal of all organic contents. Twenty-one 28S-lineages were further split by *cox1*, and several of these (dark blue) diverged morphologically. Such morphological divergence included occasional spine- or chain-formation, as well as large differences in cell size. Given that such morphological variation generally coincides with species-boundaries in diatoms[2,3], including *P. borealis*[4], all lineages delimited by *cox1* were accepted as species. The blue circle thus indicates the consensus species delimitation used for the downstream analyses. Statistical support values (BEAST posterior probabilities/IQ-TREE ultrafast bootstraps) are indicated by the colored circles on the nodes in the phylogeny: black = high support (≥ 95), and grey = moderate support (≥ 90). **b**, LM pictures showing the morphological variation between the closely related *cox1*-lineages indicated in dark blue in **a**. All micrographs in **a** and **b** have been selected from a series of ten micrographs taken from each strain: all figures are representative for the corresponding strain/species.

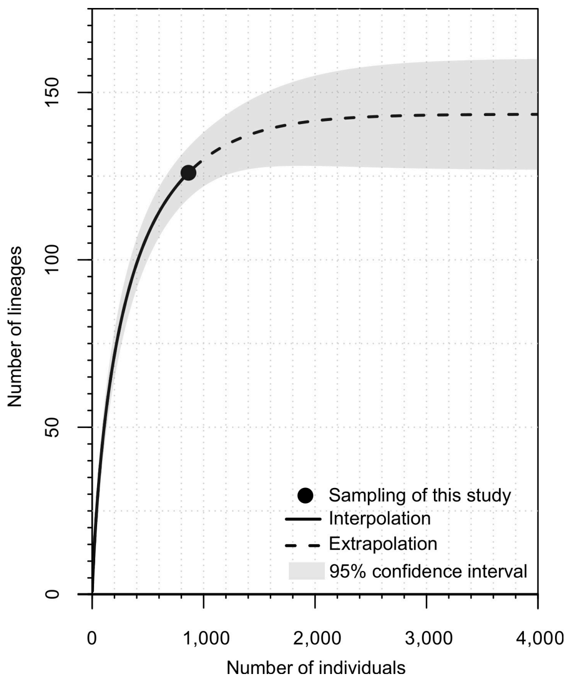

Supplementary Figure 4 | Individual-based interpolation (rarefaction) and extrapolation of the number of *P. borealis* species. The graph shows the number of detected and expected species within the investigated samples as well as the 95% confidence interval (grey area). The figure thus indicates the completeness of our culture effort. Source data are provided as a Source Data file.

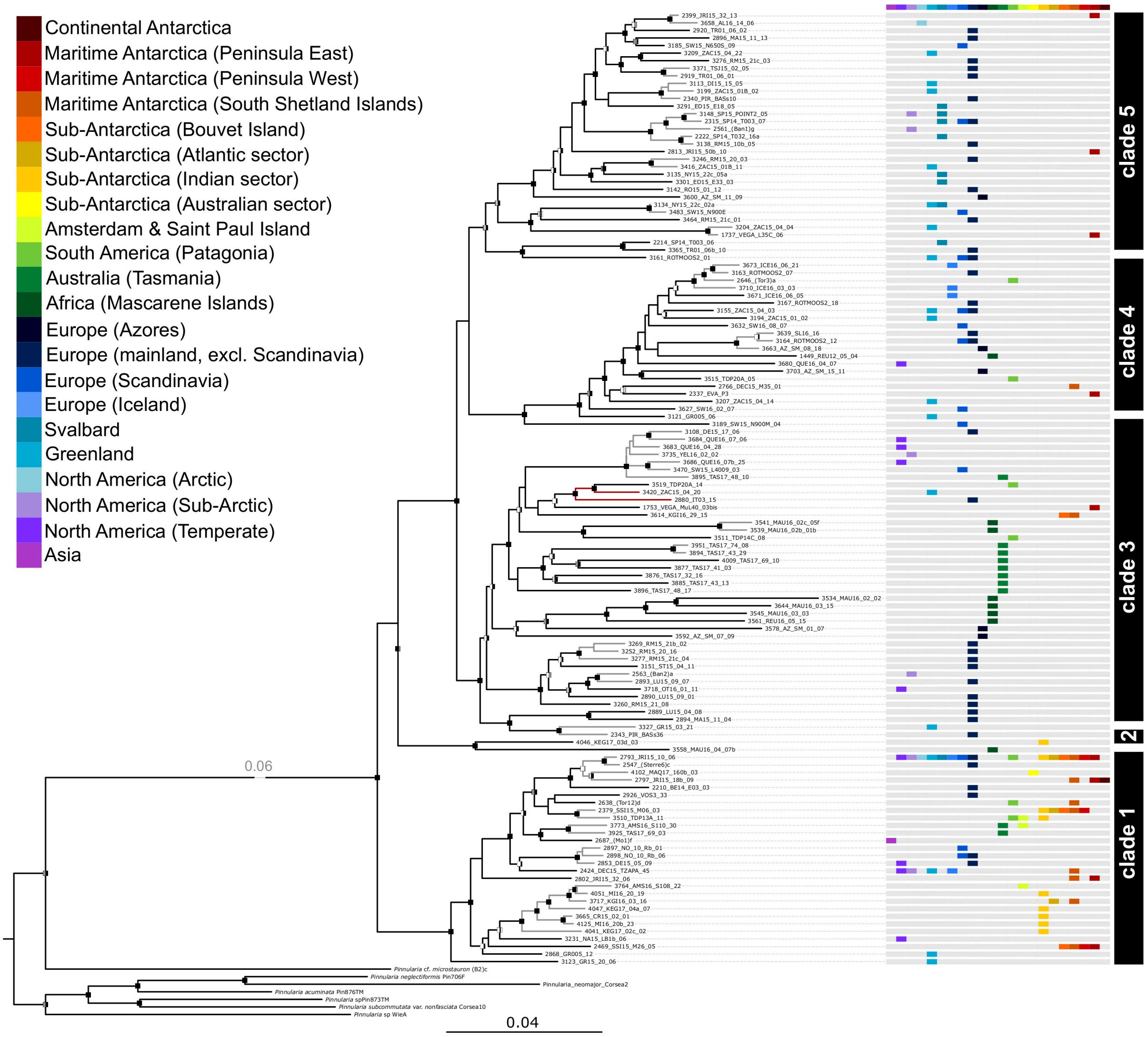

Supplementary Figure 5 | Maximum likelihood phylogeny (RAxML) based on the concatenated dataset of six markers (18S, 28S, *cox1*, *psbC*, *psbA*, and *rbcL*) revealing the presence of five major clades. One representative per consensus lineage (species) identified via the automated molecular species delimitation analyses was included in the analysis. Neighboring branches indicated in grey represent *cox1*-lineages that were lumped in the species delimitation of 28S. One 28S-lineage is paraphyletic in the concatenated phylogeny (clade 3: indicated in red). The geographic distributions of all species are indicated by the color scale (corresponding with the order in the legend). Statistical support values are indicated by the colored squares, from left to right: RAxML bootstrap (BS1), IQ-TREE non-parametric bootstrap (BS2), IQ-TREE UltraFast bootstrap (UFB), and BEAST posterior probabilities (PP). Black = high support (≥ 80 for BS1 and BS2, and ≥ 95 for UFB and PP), and grey = moderate support (≥ 70 for BS1 and BS2, and ≥ 90 for UFB and PP). The scale bar represents the ML scale of the RAxML phylogeny.

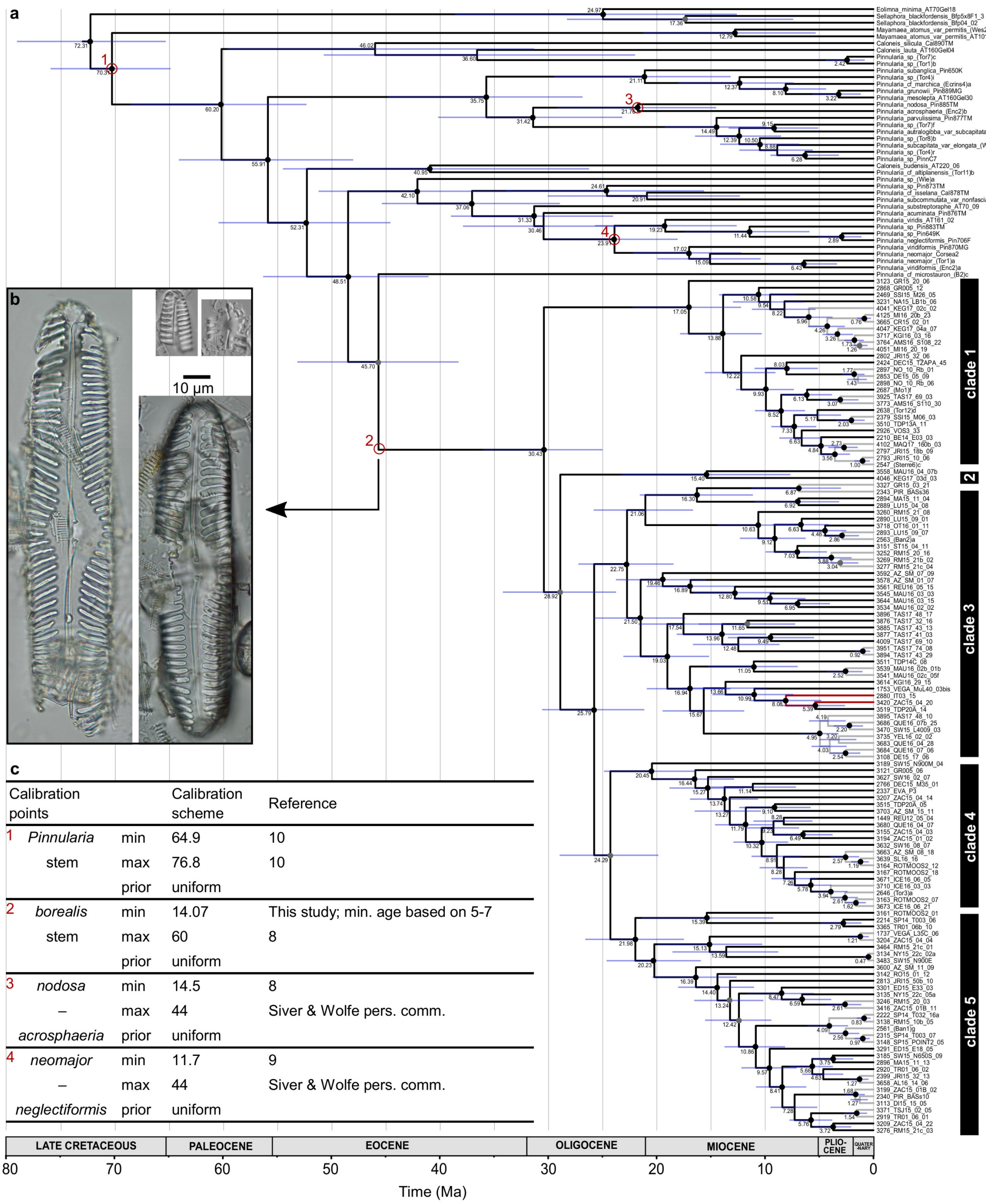

Calibration points		Calibration scheme	Reference
1 *Pinnularia* stem	min	64.9	10
	max	76.8	10
	prior	uniform	
2 *borealis* stem	min	14.07	This study; min. age based on 5-7
	max	60	8
	prior	uniform	
3 *nodosa* – *acrosphaeria*	min	14.5	8
	max	44	Siver & Wolfe pers. comm.
	prior	uniform	
4 *neomajor* – *neglectiformis*	min	11.7	9
	max	44	Siver & Wolfe pers. comm.
	prior	uniform	

Supplementary Figure 6 | Molecular time-calibration of the *P. borealis* complex. a, Time-calibrated phylogeny. Values at the nodes represent mean node ages. The grey bars are the 95% HPD (highest probability density) age-intervals. The five major clades of *P. borealis* are indicated. Neighboring branches indicated in grey represent *cox1*-lineages that were lumped in the species delimitation of 28S. One 28S-lineage is paraphyletic in the concatenated phylogeny (clade 3: indicated in red). Colored circles represent BEAST posterior probabilities: black ≥ 95, and grey ≥ 85. The constrained nodes in the molecular clock analysis are indicated with numbered red circles. **b**, Light microscopy micrographs of middle Miocene fossils (Friis Hills, McMurdo Dry Valleys, Continental Antarctica) representing two distinct morphospecies belonging to the *P. borealis* complex, and which were used as time-constraint for the origin of the *P. borealis* complex. These fossils were found in fossil lake sediments and have an estimated age of ~14–19 Ma (million years ago)[5–7]. The micrographs show all fossils of the *P. borealis* complex that were observed in the Miocene material. Scale bar = 10 µm. **c**, Table showing the calibration strategy with indication of the references for each calibration point[5–10]. The calibration points are numbered according to the phylogeny (**a**). All ages are indicated in Ma. Source data are provided as a Source Data file.

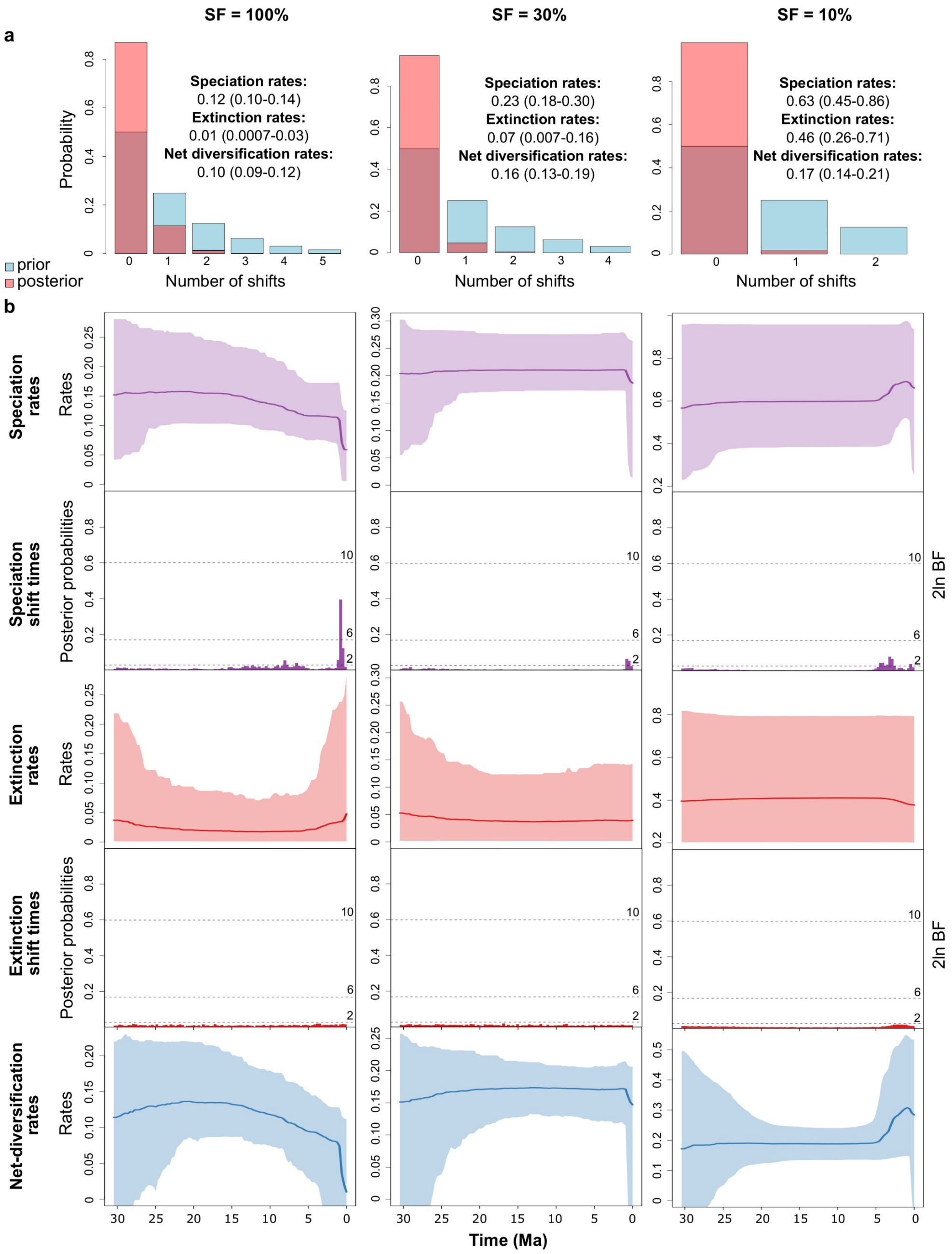

Supplementary Figure 7 | Results of the BAMM and CoMET (TESS) analyses on the diversification in the *P. borealis* complex for three different sample fractions (SF). a, Prior and posterior distributions for a number of diversification-rate shifts in the *P. borealis* phylogeny, as recovered by BAMM. For each SF, the mean speciation, extinction and net diversification rates and their 90% highest probability density (HPD) intervals as estimated by BAMM are given. **b**, Results of the CoMET analyses showing the changes in posterior mean (line) and 95% confidence intervals (colored area) of the speciation, (relative) extinction, and net-diversification rates in the *P. borealis* complex through time, as well as the shift times for the evolutionary events. All analyses were run with hyper-priors assuming one mass extinction event and one diversification rate shift. For both **a** and **b**, three different sample fractions are shown, representing scenarios where sampling is complete (100%) or incomplete (30% or 10% of the actual number of species is present in the phylogeny). Since the species accumulation curve (Fig. 2) indicates that our sampling of *P. borealis* is incomplete, the 100% sampling fraction represents an absolute baseline for *P. borealis* diversification, and provides a minimum boundary for the estimated rates. Legend: BF = Bayes factor. All rates are indicated in 'events per lineage per million years'.

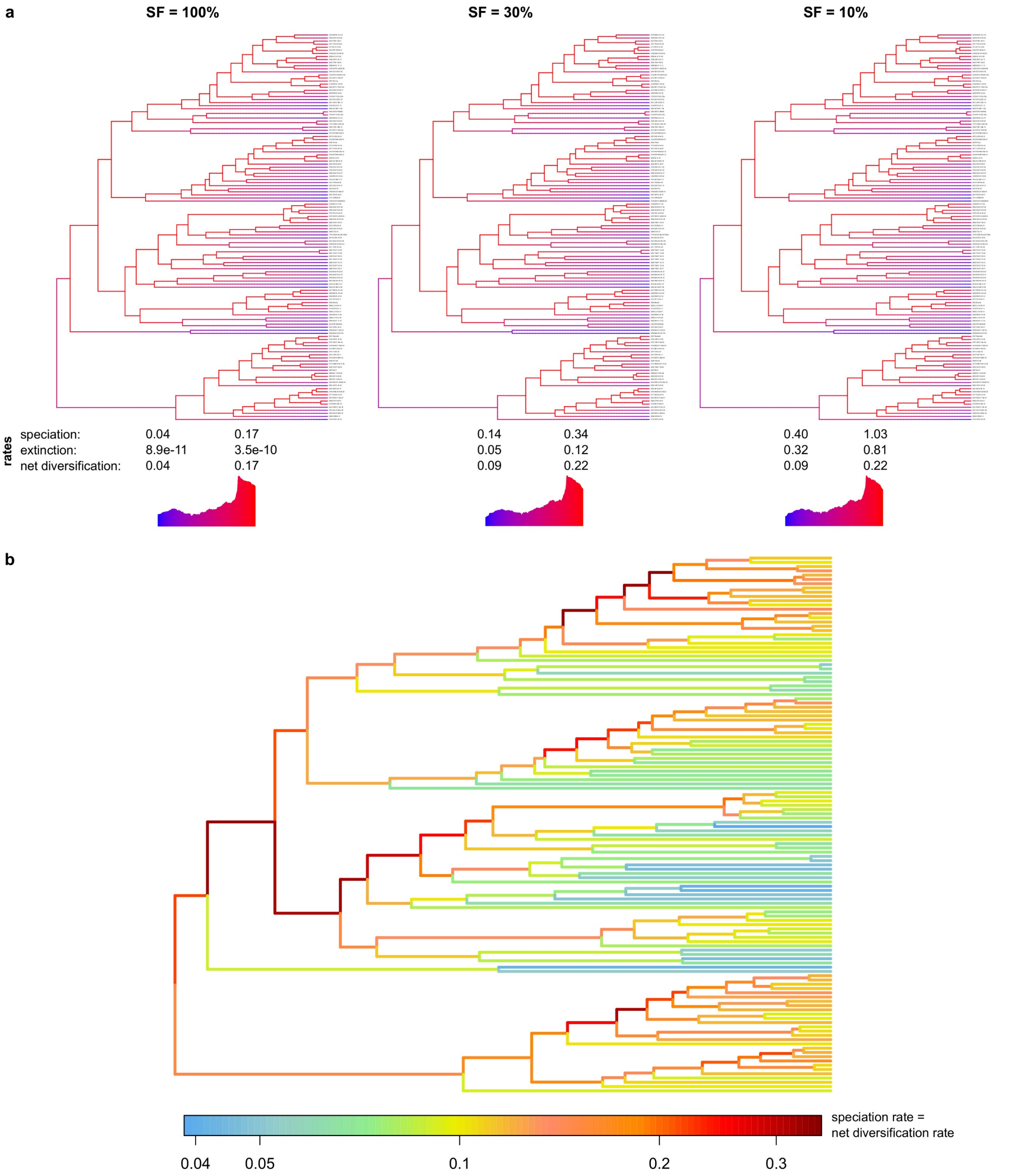

Supplementary Figure 8 | Results of the MiSSE and ClaDS0 analyses on the diversification in the *P. borealis* complex. a, Results of the MiSSE analyses for three different sampling fractions (SF). The colored scale below the phylogenies indicates the range and density of the speciation, extinction and net diversification rates throughout the phylogenies. The values above the scale indicate the minimum – maximum range of all estimated rates. Note that the ratio between speciation and extinction remains constant throughout the phylogeny because the extinction fraction was not varied in the analyses. **b**, Results of the ClaDS0 analysis (100% SF). The color scale below the phylogeny corresponds with the colors of the branches in the tree, showing changes in speciation/net diversification rate. A ClaDS0 analysis assumes zero extinction rates, so that the modelled speciation rates equal net diversification rates. All rates are indicated in 'events per lineage per million years'.

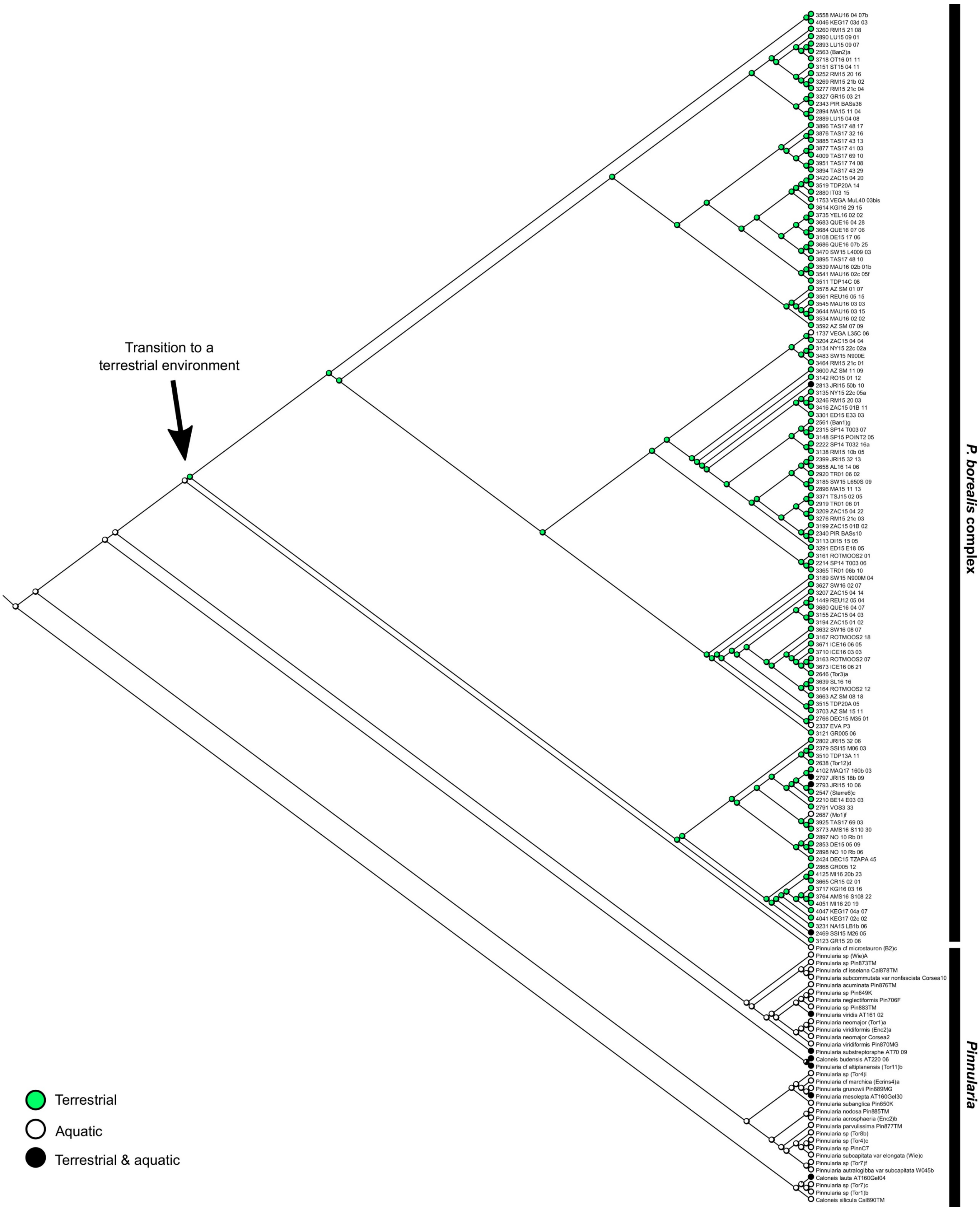

Supplementary Figure 9 | Maximum Likelihood Ancestral habitat reconstruction of the genus *Pinnularia*, including the *P. borealis* complex. ‘Aquatic habitat’ comprises submerged environments, whereas ‘terrestrial habitat’ includes both wet, moist and dry soils and mosses. The time-calibrated phylogeny (Fig. 2, Supplementary Fig. 6) of the *P. borealis* complex was used as input for the analysis. The habitat type identification of each species was based on the habitat type of all individual strains incorporated in the phylogeny. The presumed aquatic species *P. catenaborealis* is referred to as reference strain EVA_P3. Source data are provided as a Source Data file.

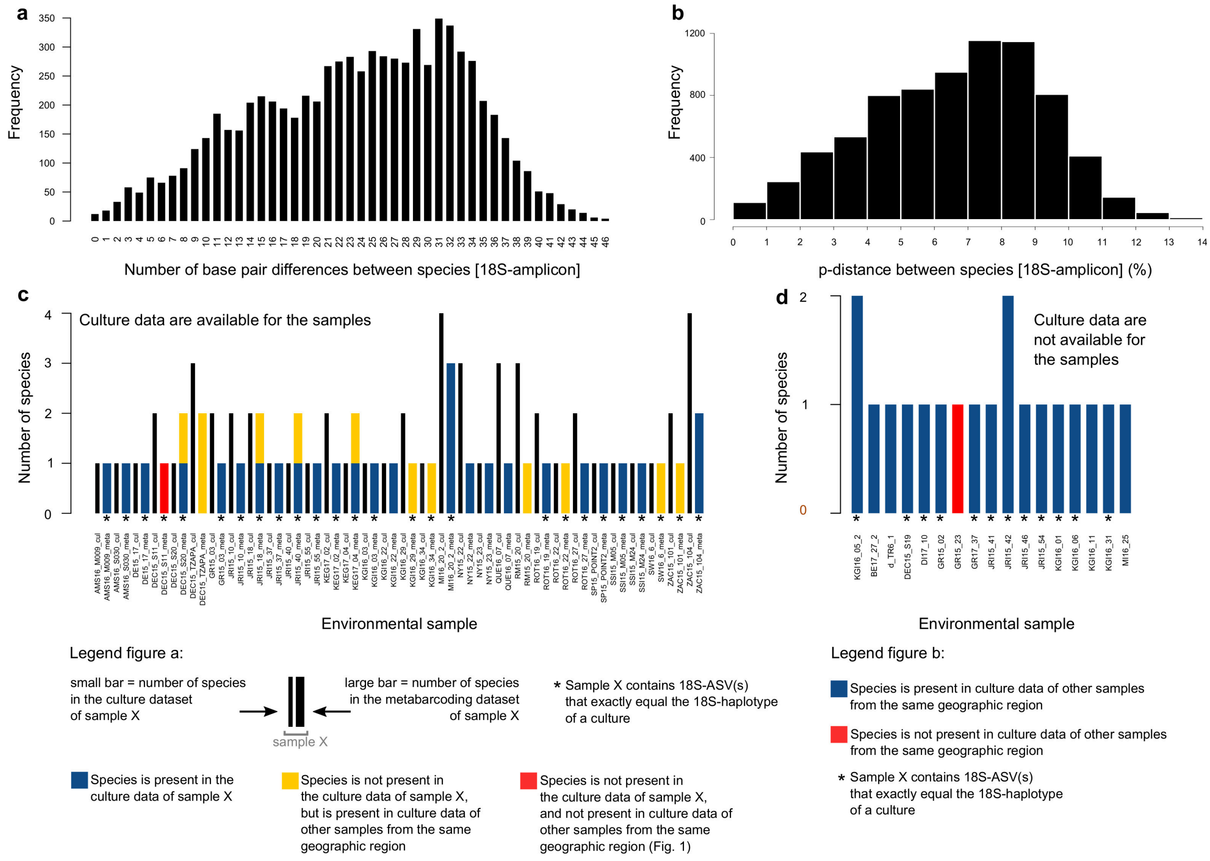

e

ASV	Species ID mothur algorithm	Region	Diff. (bp)	ASV	Species ID mothur algorithm	Region	Diff. (bp)
ASV_416	AMS16_S108_22	Sub-Antarctica	0	ASV_8394	JRI15_10_06_or_Sterre6c	Antarctica	4
ASV_1181	AMS16_S108_22	Sub-Antarctica	1	ASV_702	JRI15_10_06_or_Sterre6c	Antarctica	2
ASV_1431	AMS16_S108_22	Sub-Antarctica	1	ASV_1479	JRI15_32_06	Antarctica	0
ASV_1926	AMS16_S108_22	Sub-Antarctica	1	ASV_7466	JRI15_32_06	Antarctica	4
ASV_2369	AMS16_S108_22	Sub-Antarctica	1	ASV_1929	JRI15_50b_10	Antarctica	0
ASV_2370	AMS16_S108_22	Sub-Antarctica	1	ASV_5191	KEG17_02c_02	Sub-Antarctica	0
ASV_2533	AMS16_S108_22	Sub-Antarctica	1	ASV_1171	KEG17_03d_03	Sub-Antarctica	1
ASV_2968	AMS16_S108_22	Sub-Antarctica	1	ASV_2758	KEG17_04a_07	Sub-Antarctica	0
ASV_3043	AMS16_S108_22	Sub-Antarctica	1	ASV_457	KGI16_03_16_or_MI16_20_19	(Sub-)Antarctica	0
ASV_3229	DE15_17_06	Palaearctic (excl. Arctic)	0	ASV_3355	KGI16_03_16_or_MI16_20_19	Sub-Antarctica	1
ASV_5922	DE15_17_06	Palaearctic (excl. Arctic)	5	ASV_5117	KGI16_03_16_or_MI16_20_19 †	Arctic †	2
ASV_173	DEC15_M35_01	Antarctica	0	ASV_940	MI16_20b_23	Sub-Antarctica	0
ASV_424	DEC15_M35_01	Antarctica	1	ASV_3732	MI16_20b_23	Sub-Antarctica	2 *
ASV_790	DEC15_M35_01	Antarctica	2	ASV_7261	MI16_20b_23	Sub-Antarctica	2 *
ASV_499	DEC15_M35_01	Antarctica	2	ASV_6254	NY15_22c_02a	Arctic	0
ASV_3252	GR15_03_21	Arctic	0	ASV_2511	SP15_POINT2_05_or_SP14_T003_07	Arctic	0
ASV_155	JRI15_10_06_or_Sterre6c	Antarctica & Arctic	0	ASV_5893	SP15_POINT2_05_or_SP14_T003_07	Palaearctic (excl. Arctic)	2
ASV_2940	JRI15_10_06_or_Sterre6c	Antarctica	1	ASV_1417	SSI15_M06_03	(Sub-)Antarctica	0
ASV_5444	JRI15_10_06_or_Sterre6c	Arctic	1	ASV_7714	SSI15_M26_05	Antarctica	4
ASV_6455	JRI15_10_06_or_Sterre6c	Arctic	1	ASV_4606	(Tor12)d †	Antarctica †	0
ASV_6612	JRI15_10_06_or_Sterre6c	Nearctic	2	ASV_8036	ZAC15_04_03	Arctic	6
ASV_1139	JRI15_10_06_or_Sterre6c	Antartcia	3				

* ASV contains large deletions, and non-alignable tail up to 20 bp

† This species was not found in this geographic region in the culture dataset

Supplementary Figure 10 | Detection and identification of *P. borealis* ASVs (18S) in the metabarcoding dataset. a–b, Frequency distribution showing the number of base pair differences (**a**), and the p-distance in percentage (**b**) between species. These calculations are based on the 18S-alignment of the culture data, taking only the amplicon into account. Source data are provided as a Source Data file. **c–d,** Figures showing the number of *P. borealis* species detected by metabarcoding/cultures for all environmental samples for which both culture material and metabarcoding data were available (**c**), and for which only metabarcoding data were available (**d**). **e,** Table showing the identification of all *P. borealis* ASVs (based on Mothur), and the number of base pair (bp) differences with the 18S-reference sequences of the cultures. When species had identical 18S-haplotypes for the amplicon, both *P. borealis* species are listed. For each ASV, the geographic region in which it was found is indicated. For the geographic distributions of individual species as used in BioGeoBEARS, only those ASVs which were identical to reference sequences were taken into account.

a

	LnL*	# parameters†	*d*‡	*e*§	*j*‖	*x*¶	AIC
DEC+*j* +*x*	-287.1	4	0.0008	1.0e-12	0.025	-1.00	582.6
DIVALIKE+*j* +*x*	-296.7	4	0.0010	1.0e-12	0.022	-1.07	601.6
BAYAREALIKE+*j* +*x*	-300.4	4	0.0007	1.0e-07	0.028	-1.13	609.2
DEC+*j*	-313.8	3	0.0008	1.0e-12	0.061	0	633.8
DIVALIKE+*j*	-317.1	3	0.0032	1.0e-12	0.057	0	640.3
BAYAREALIKE+*j*	-322.7	3	0.0025	1.0e-07	0.067	0	651.6
DIVALIKE+*x*	-353.6	3	0.0030	0.0018	0	-1.04	713.5
DEC+*x*	-353.7	3	0.0023	0.0079	0	-1.05	713.6
DEC	-369.3	2	0.0068	0.0063	0	0	742.7
DIVALIKE	-373.0	2	0.0088	1.0e-12	0	0	750.0
BAYAREALIKE+*x*	-396.9	3	0.0018	0.0980	0	-1.13	799.9
BAYAREALIKE	-414.7	2	0.0100	0.0830	0	0	833.5

* Log likelihood
† Number of parameters in each model
‡ Range expansion ('dispersal') ML parameter estimate
§ Range contraction ('extinction') ML parameter estimate
|| Founder-event ML parameter estimate
¶ Distance model ML parameter estimate

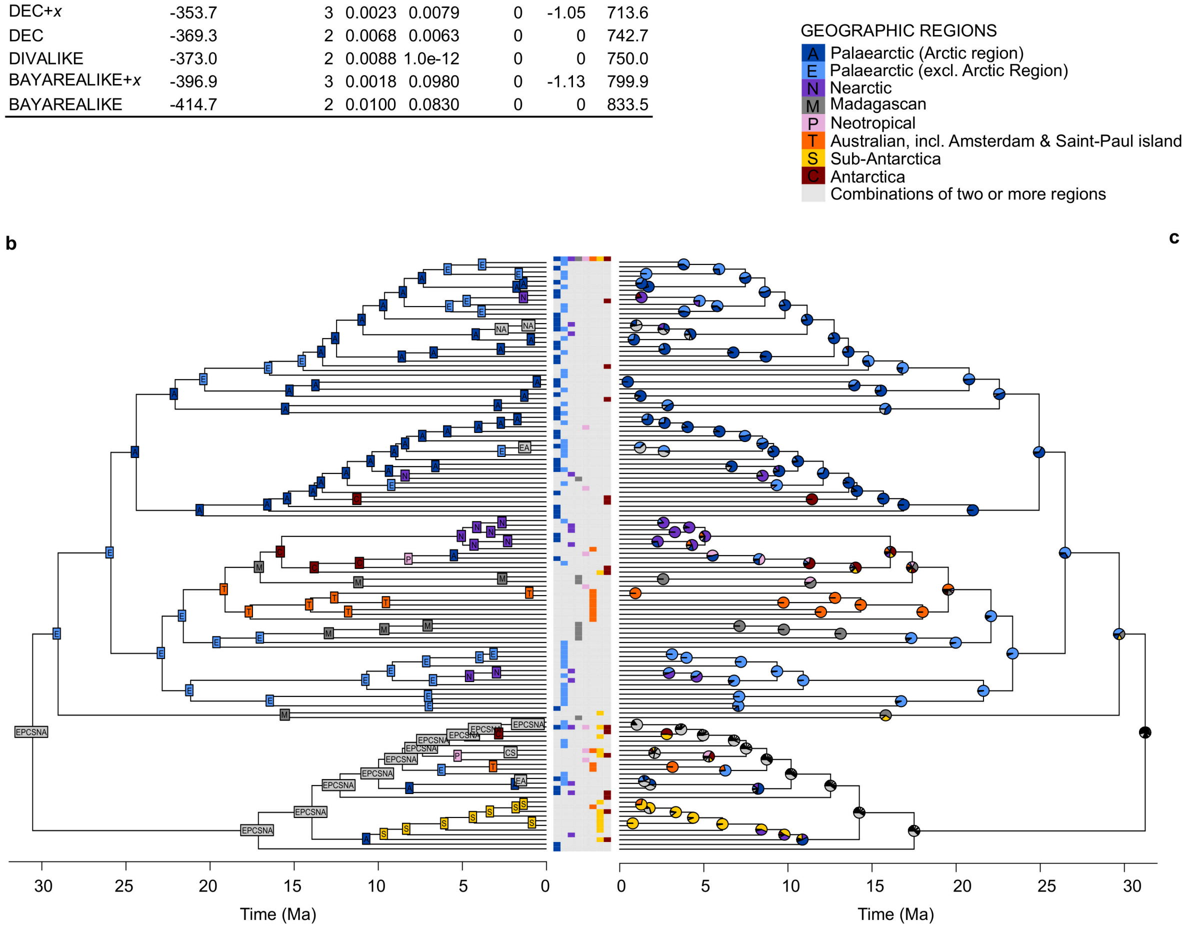

Supplementary Figure 11 | Analysis on the historical biogeography of the *P. borealis* complex in BioGeoBEARS. a, Table showing the results of the BioGeoBEARS analyses. The different models are ranked according to their AIC value. The parameters *j* and *x* were fixed to zero in the models in which they were not included. The best model (lowest AIC) is underlined. The top three favored models all incorporate the founder event estimate (+*j*) and the distance model parameter (+*x*). This indicates that range expansion/contraction alone (anagenetic processes) cannot accommodate for changes in geographic distributions, and that between-region dispersal is function of the geographic distance, i.e. dispersal probability decreases with increasing geographic distance. **b-c**, Figures showing the results of the favored model (DEC+*j*+*x*): the single-most-probable biogeographic range at each node in the phylogeny (**b**), and the probabilities of each possible geographical range at each node in the phylogeny (**c**). The extant geographic distribution of each species is indicated by the colored squares in the middle. The time-calibrated phylogeny of the *P. borealis* complex (Fig. 2, Supplementary Fig. 6) was used as input for the analyses. Source data are provided as a Source Data file.

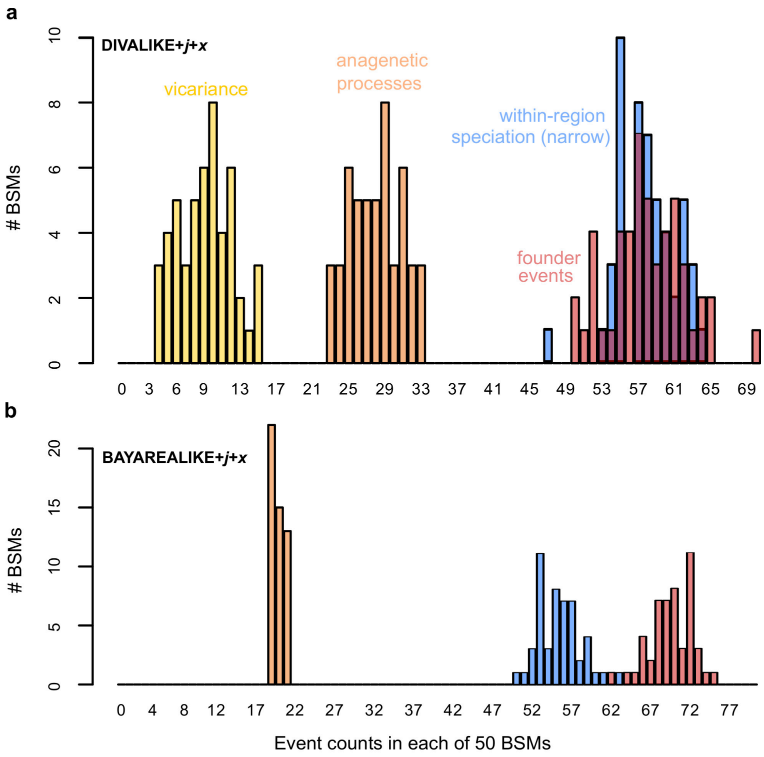

Supplementary Figure 12 | Probabilities of different biogeographic processes at each node in the *P. borealis* phylogeny, as assessed by Biogeographic Stochastic Mapping (BSM) in BioGeoBEARS. a, Histograms showing the frequency distribution of the four different types of historical biogeographical processes included in the second-best model in BioGeoBEARS (DIVALIKE+*j*+*x*) in 50 BSMs. **b**, Histograms showing the frequency distribution of the three different types of historical biogeographical processes included in the third best model in BioGeoBEARS (BAYAREALIKE+*j*+*x*) in 50 BSMs. In both **a** and **b**, the x-axis gives the number of events, and the y-axis gives the number of BSMs in which a certain number of biogeographic processes was observed.

a

Population strata	Covariance (σ)	Covariance (%)	Phi (ϕ)	*P*-value
Non-clone-corrected data				
within samples	473	54.1	0.797	**0.001 (0.003)**
between samples, within hemispheres	224	25.6	0.557	**0.001 (0.003)**
between hemispheres	178	20.3	0.541	**0.001 (0.003)**
Clone-corrected data				
within regions	396	47.6	0.307	0.289 (0.867)
between regions, within hemispheres	-140	-16.8	-0.321	0.789 (1.000)
between hemispheres	557	69.3	0.476	**0.001 (0.003)**

b

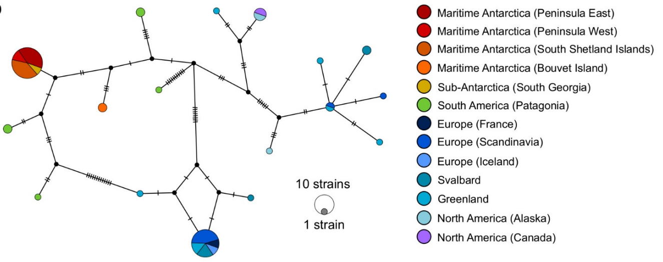

c

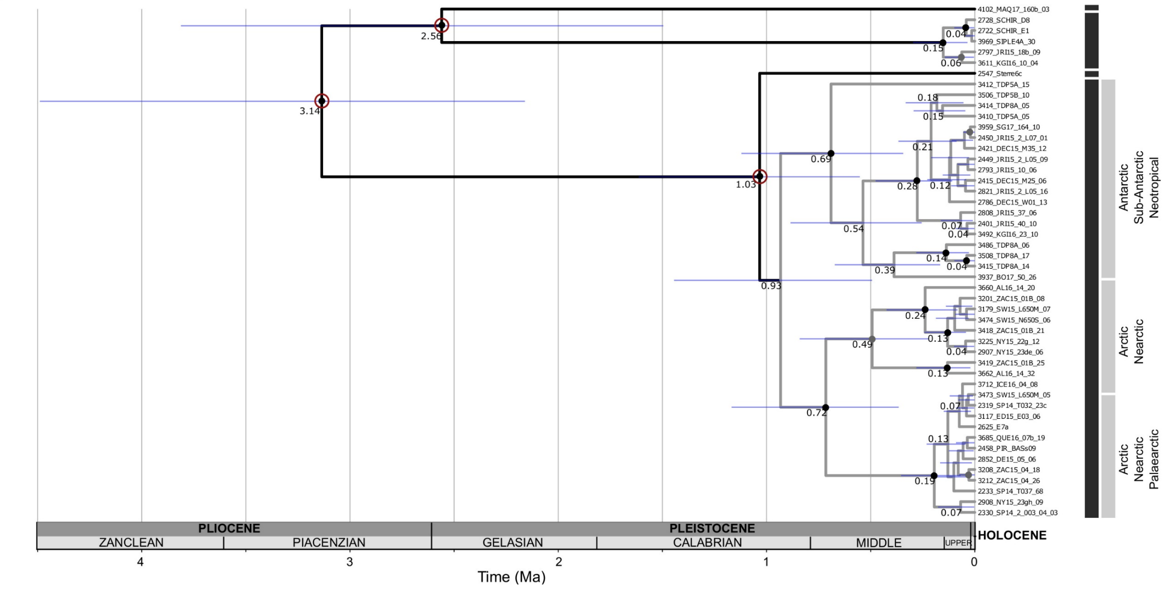

Supplementary Figure 13 | Phylogeography of the most widely distributed and common *P. borealis* species in this dataset (reference strain JRI15_10_06). **a**, Results of AMOVA and randomization tests on *P. borealis* species JRI15_10_06. *P*-values within brackets have been adjusted for multiple testing using Bonferroni correction. Significant *P*-values are indicated in bold. A sample refers to the original environmental sample from which the strains were isolated. **b**, Haplotype network (TCS) of the *cox1* gene of *P. borealis* lineage JRI15_10_06. The color code is identical to Supplementary Fig. 5. Haplotypes of the Northern (blue – purple) and Southern Hemisphere (red – yellow – green) are clearly separated, indicative of inter-hemisphere population structuring. There is also evidence for the existence of two distinct groups in the Northern Hemisphere. **c**, Time-calibrated phylogeny of four closely related lineages of the *P. borealis* species complex, based on a three-gene alignment (28S, *cox1*, *rbcL*). Values at the nodes represent mean node ages. The grey bars are the 95% HPD (highest probability density) age-intervals. Constrained nodes are indicated with red circles. Colored circles represent BEAST posterior probabilities: black ≥ 95, and grey ≥ 85. The dark grey bars represent the consensus lineages as delimited by *cox1* – the light grey bars show the three main clusters within *P. borealis* species JRI15_10_06. Most sequences represent multiple strains with the same haplotype. Note that the position of *P. borealis* lineage (Sterre6)c is uncertain, relative to the three clusters within JRI15_10_06, as the split between (Sterre6)c and the clades within JRI15_10_06 has a low BI support value. (Sterre6)c was delimited by all distance- and tree-based methods of automated molecular species delimitation in the *cox1*-dataset. This low support value could indicate problems with incomplete lineage sorting between closely related species. Alternatively (Sterre6)c could be conspecific with JRI15_10_06, or several of the clades within JRI15_10_06 are subject to ongoing speciation or represent yet unidentified species. The latter was also suggested by sGMYC which delimited the three clades within JRI15_10_06 as species in the *cox1*-dataset (Supplementary Fig. 2). Source data are provided as a Source Data file.

Supplementary Tables.

Supplementary Table 1 | PCR and sequencing primers, PCR conditions, and general characteristics of the six-marker alignment. a, Oligonucleotide primer sequences used for PCR and/or sequencing reactions[11–21]. **b**, PCR conditions for the different primer pairs. **c**, Overview of the alignment characteristics of the 126 consensus lineages (species) obtained from the automated molecular species delimitation. For each species, one reference strain was selected. The dataset was incomplete for 18S, *psb*A, and *psbC*. For the non-coding genes 18S and 28S, insertions that were unique for a given sequence were deleted prior to the calculations.

a

Marker	Primer	Primer sequence (5'–3')	Reference
18S	ELW528	CGGTAATTCCAGCTCC (→)	11
18S	P4	TGATCCTTCYGCAGGTTCAC (←)	12,15
18S	ELW960	TTTGACTCAACACGGG (→)	11
18S	ELW1055	CGGCCATGCACCAC (←)	11
18S	D514	TCCAGCTCCAATAGCGTA (→)	16
18S	D1069	TCTTTAAGTTTCAGCCTTGC (←)	16
28S	DIR-f	ACCCGCTGAATTTAAGCATA (→)	17
28S	T24U	SCWCTAATCATTCGCTTTACC (←)	18
28S	D2C-r	CCTTGGTCCGTGTTTCAAGA (→)	17
28S	T16N	AMAAGTACCRYGAGGGAAAG (←)	18
28S	PBLSU1F	TGAAAMGGAAGCRAACRRASSTAGTG (←)	This study
28S	PBLSU1R	GACCATWWTCCCRARGGATBGRGGC (←)	This study
28S	PBLSU2F	TGGAAYAAWAYAGCANAGARGGTG (→)	This study
28S	PBLSU2R	TTCWAGACGGGRCAAACAAABCTA (←)	This study
cox1	pB1*	GCWACWACRTARTAWGTRTCRTG (→)	14
cox1	pC1*	TGGTTNTTYTCNACNAAYCAYAARGAYAT (←)	14
cox1	PBORcox1F	GTNACNGGNCAYGCDATTATHATG (→)	This study
cox1	PBORcox1R	CCNGTAGCHCCNCCNACWGTAA (←)	This study
cox1	PBORcox2F	ATGGTNATGCCNKCTYTHATWGGNGG (→)	This study
cox1	PBORcox2R	CCCCAHARNGTDGCYAACCARCT (←)	This study
cox1	PBORcox6F	TTAAGTTTTTGGTTRTTACCNCC (→)	This study
cox1	PBORcox6R	TANACTTCDGGRTGNCCRAAAAACC (←)	This study
psbA	PsbAF	ATGACTGCTACTTTAGAAAGACG (→)	19
psbA	PsbAR1	GCTAAATCTARWGGGAAGTTGTG (←)	19
psbC	psbC22	CGTGGTGATACATAGTTA (→)	20
psbC	psbC1154	GCDCAYGCTGGYTTAATGG (←)	20
rbcL	DPrbcL1-F	AAGGAGAAATHAATGTCT (→)	21
rbcL	DPrbcL7-R	AACAACCTTGTGTAAGTCTC (←)	21
rbcL	RbcL13-F	CGTTTAGAAGATATGCGTATTC (→)	13
rbcL	RbcL-17R	TGACCAATTGTACCACC (←)	21

* Only used for primer development, but not for routine PCR amplification or sequencing
The arrows indicate the primer direction: forward (→) and reverse (←)
The colored bars indicates the primer pairs: grey (PCR amplification and sequencing) or black (only sequencing)

b

	D1–D3 28S*	V4–V9 18S	*cox1**	*psbA*	*psbC*	*rbcL*
Initial denaturation	95°C (5')	94°C (3')	95°C (3')	94°C (3')	95°C (3')	94°C (3')
Annealing						
Denaturation	94°C (1')	94°C (1')	95°C (30 s)	94°C (1')	95°C (1')	94°C (1')
Annealing	55°C (1')	55°C (1')	55°C (1')	46°C (1')	55.5°C (1')	55°C (1')
Extension	74°C (1')	72°C (1.5')	72°C (1.5')	72°C (2')	72°C (1')	72°C (1.5')
# cycles	35	40	35	35	35	40
Final elongation	72°C (10')	72°C (10')	72°C (5')	72°C (10')	72°C (10')	72°C (5')

* For both 28S and *cox1* the same PCR conditions were used for all primer pairs, except for PBORcox6F/6R for which 51 °C was used as annealing temperature

c

	D1–D3 28S	V4–V9 18S	*cox1*	*psbA*	*psbC*	*rbcL*
#* sequences	126	124	126	38	48	126
alignment length	1088 (938)§	1124 (1105)§	726	896	1014	1358
# variable positions	369	168	318	48	130	263
# PI† positions	291	129	292	22	67	183
p-distance (mean±SD‡)	0.094±0.026	0.031±0.012	0.129±0.022	0.007±0.003	0.018±0.010	0.023±0.008
p-distance (min-max)	0.0–0.227	0.0–0.072	0.017–0.199	0.0–0.016	0.0–0.038	0.0–0.042
# differences (mean±SD)	79.8±21.3	31.2±11.9	87.6±17.7	5.6±2.3	6.4±17.2	9.9±29.8
# differences (min-max)	0–153	0–64	11–131	0–14	0–30	0–56

* Number of
† Parsimony informative
‡ Standard deviation
§ For the ribosomal genes, the number between brackets gives the length of the longest sequence when not aligned (i.e. without introduction of insertions in the loop regions)

Supplementary Table 2 | Strain list of the outgroup sequences (*Pinnularia*, *Caloneis*, *Mayamaea*, *Sellaphora* and *Eolimna*) used in this study. For each strain, the reference towards the original study is indicated (refs[8,22,23]). N.A. = not available.

Strain	Species	28S	18S	*cox*1	*psb*A	*psb*C	*rbc*L	References
(B2)c	*Pinnularia* cf. *microstauron* (Ehrenberg) Cleve	MH707907	JN418568	JN418675	JN418703	MN319651	JN418638	8; this study
(Bfp04)02	*Sellaphora blackfordensis* Mann & Droop	MN319622	N.A.	N.A.	N.A.	N.A.	JN418674	8; this study
(Bfp5x8)F1-3	*Sellaphora blackfordensis* Mann & Droop	MN319640	JN418599	JN418699	JN418734	N.A.	JN418669	8; this study
(Ecrins4)a	*Pinnularia* cf. *marchica* Schönfelder	MN319635	JN418569	JN418676	JN418704	N.A.	JN418639	8; this study
(Enc2)a	*Pinnularia viridiformis* Krammer	MN319633	JN418574	JN418679	JN418709	N.A.	JN418644	8; this study
(Enc2)b	*Pinnularia acrosphaeria* Smith	MN319625	N.A.	JN418701	JN418737	N.A.	JN418672	8; this study
(Tor1)a	*Pinnularia neomajor* Krammer	MN319641	JN418571	JN418677	JN418706	N.A.	JN418641	8; this study
(Tor1)b	*Pinnularia* sp. (divergens-group)	MN319629	JN418572	N.A.	JN418707	N.A.	JN418642	8; this study
(Tor11)b	*Pinnularia* cf. *altiplanensis* Lange-Bertalot	MN319624	JN418573	JN418678	JN418708	N.A.	JN418643	8; this study
(Tor4)i	*Pinnularia* sp.	MN319631	JN418580	JN418683	JN418715	N.A.	JN418650	8; this study
(Tor4)r	*Pinnularia* sp.	MN319643	JN418581	JN418684	JN418716	N.A.	JN418651	8; this study
(Tor7)c	*Pinnularia* sp. (divergens-group)	MN319627	JN418582	N.A.	JN418717	N.A.	JN418652	8; this study
(Tor7)f	*Pinnularia* sp. (gibba-group)	MN319644	JN418576	JN418680	JN418711	N.A.	JN418646	8; this study
(Tor8)b	*Pinnularia* sp. (gibba-group)	MN319628	JN418577	JN418681	JN418712	N.A.	JN418647	8; this study
(W045)b	*Pinnularia australogibba* var. *subcapitata* Van de Vijver, Chat	MN319621	JN418738	JN418702	JN418738	N.A.	JN418673	8; this study
(Wes2)f	*Mayamaea atomus* var *permitis* (Hustedt) Lange-Bertalot	MN319620	JN418600	JN418700	JN418735	N.A.	JN418670	8; this study
(Wie)a	*Pinnularia* sp.	MH707935	JN418578	N.A.	JN418713	MN319652	JN418648	8; this study
(Wie)c	*Pinnularia subcapitata* var. *elongata* Krammer	MN319619	JN418579	JN418682	JN418714	N.A.	JN418649	8; this study
AT-101Gel04	*Mayamaea atomus* var. *permitis* (Hustedt) Lange-Bertalot	AM710524	AM501969	N.A.	N.A.	N.A.	AM710435	23
AT-160Gel04	*Caloneis lauta* Carter & Bailey-Watts	AM710595	AM502039	N.A.	N.A.	N.A.	AM710506	23
AT-160Gel30	*Pinnularia mesolepta* (Ehrenberg) Smith	AM710550	AM501994	N.A.	N.A.	N.A.	AM710461	22
AT-161.02	*Pinnularia viridis* (Nitzsch) Ehrenberg	N.A.	AM502023	N.A.	N.A.	N.A.	AM710490	22
AT-220.06	*Caloneis budensis* (Grunow) Krammer	AM710559	AM502003	N.A.	N.A.	N.A.	AM710470	23
AT-70.09	*Pinnularia substreptoraphe* Krammer	AM710592	AM502036	N.A.	N.A.	N.A.	AM710503	22
AT-70Gel18	*Eolimna minima* (Grunow) Lange-Bertalot	AM710516	AM501962	N.A.	N.A.	N.A.	AM710427	23
Cal878TM	*Pinnularia* cf. *isselana* Krammer	MN319639	JN418594	JN418694	JN418729	N.A.	JN418664	8; this study
Cal890TM	*Caloneis silicula* (Ehrenberg) Cleve	MN319637	JN418593	N.A.	N.A.	N.A.	JN418663	8; this study
Corsea10	*Pinnularia subcommutata* var. *nonfasciata* Krammer	MH707925	JN418584	JN418686	JN418719	N.A.	JN418654	8; this study
Corsea2	*Pinnularia neomajor* Krammer	MH707898	JN418585	JN418687	JN418720	N.A.	JN418655	8; this study
Pin649K	*Pinnularia* sp. (subcommutata-group)	MN319632	JN418595	JN418695	JN418730	N.A.	JN418665	8; this study
Pin650K	*Pinnularia subanglica* Krammer	MN319634	JN418598	JN418698	JN418733	N.A.	JN418668	8; this study
Pin706F	*Pinnularia neglectiformis* Krammer	MH707902	JN418596	JN418696	JN418731	N.A.	JN418666	8; this study
Pin870MG	*Pinnularia viridiformis* Krammer	MN319636	JN418589	JN418691	JN418724	N.A.	JN418659	8; this study
Pin873TM	*Pinnularia* sp.	MH707900	JN418590	JN418692	JN418725	N.A.	JN418660	8; this study
Pin876TM	*Pinnularia acuminata* Smith	MH707896	JN418597	JN418697	JN418732	N.A.	JN418667	8; this study
Pin877TM	*Pinnularia parvulissima* Krammer	MN319626	JN418591	JN418693	JN418726	N.A.	JN418661	8; this study
Pin883TM	*Pinnularia* sp. (subcommutata-group)	MN319638	JN418586	JN418688	JN418721	N.A.	JN418656	8; this study
Pin885TM	*Pinnularia nodosa* (Ehrenberg) Smith	MN319623	JN418587	JN418689	JN418722	N.A.	JN418657	8; this study
Pin889MG	*Pinnularia grunowii* Krammer	MN319630	JN418588	JN418690	JN418723	N.A.	JN418658	8; this study
PinnC7	*Pinnularia* sp.	MN662533	JN418583	JN418685	JN418718	N.A.	JN418653	8; this study

Supplementary Table 3 | Substitution models and partition schemes used for the phylogenetic analyses. For analysis 13, standard substitution models were used in BEAST v2.5.0 using the SSM package, v1.0.1[24].

	Analysis	Gene(s)	Program	# Partitions	Substitution models & partitioning schemes
1	Automated molecular species delimitation (sGMYC)	*cox* 1	MrBayes	3	GTR+I+G (full codon partition)
2	Automated molecular species delimitation (sGMYC)	28S	MrBayes	1	GTR+I+G
3	Automated molecular species delimitation (PTP)	*cox* 1	RAxML	3	GTR+I+G (full codon partition)
4	Automated molecular species delimitation (PTP)	28S	RAxML	1	GTR+I+G
5	Two-gene haplotype phylogeny	*cox* 1, 28S	IQ-TREE	4	SYM+I+G (28S), GTR+I+G (1st codon *cox* 1), TrN+I+G (2nd codon *cox* 1), and TIM+I+G (3th codon *cox* 1)
6	Two-gene haplotype phylogeny	*cox* 1, 28S	BEAST2	4	SYM+I+G (28S), GTR+I+G (1st codon *cox* 1), TrN+I+G (2nd codon *cox* 1), and TIM+I+G (3th codon *cox* 1)
7	Single gene phylogeny	*cox* 1	IQ-TREE	3	SYM+I+G (1st codon), TrN+I+G (2nd codon), and GTR+I+G (3th codon)
8	Single gene phylogeny	28S	IQ-TREE	1	GTR+I+G
9	Single gene phylogeny	18S	IQ-TREE	1	TMP3u+I+G
10	Single gene phylogeny	*rbc* L	IQ-TREE	3	K81uf+I+G (1st codon), SYM+I+G (2nd codon), and GTR+I+G (3th codon)
11	Single gene phylogeny	*psb* A	IQ-TREE	2	F81+I (1st and 2nd codon), and HKY+G (3th codon)
12	Single gene phylogeny	*psb* C	IQ-TREE	3	TrN+I (1st codon), JC (2nd codon), and TIM+G (3th codon)
13	Six-marker phylogeny *P. borealis*	*cox* 1, 28S, 18S, *rbc* L, *psb* A, *psb* C	IQ-TREE	10	GTR+I+G (28S; 1st codon *cox* 1; 3th codon *cox* 1), TVM+I+G (18S; 3th codon *psbC* and *rbcL*), TrN+I+G (2nd codon *cox* 1 and *psb* A; 1st codon *psb* A, *psb* C, and *rbc* L), SYM+I+G (2nd codon *rbc* L), TrN+G (3th codon *psb* A), and JC (2nd codon *psb* C)
14	Six-marker phylogeny *P. borealis*	*cox* 1, 28S, 18S, *rbc* L, *psb* A, *psb* C	BEAST2	10	Same partition scheme and substitution models as analysis 13
15	Six-marker phylogeny *P. borealis*	*cox* 1, 28S, 18S, *rbc* L, *psb* A, *psb* C	RAxML	10	GTR+I+G (using the same partitions as in analyses 13 and 14)
16	Time-calibrated phylogeny *P. borealis* + *Pinnularia*	*cox* 1, 28S, 18S, *rbc* L, *psb* A, *psb* C	BEAST1	8	SYM+I+G (28S), GTR+I+G (1st codon cox1; 3th codon *cox* 1; 18S; 2nd codon of *rbc* L, *psb* A and *psb* C; 3th codon of *rbc* L, *psb* A and *psb* C), TrN+I+G (2nd codon *cox* 1), HKY+I+G (1st codon *rbc* L, *psb* A and *psb* C)

Supplementary References

1. Lynch, M. D. J. & Neufeld, J. D. Ecology and exploration of the rare biosphere. *Nat. Rev. Microbiol.* **13**, 217–229 (2015).

2. Mann, D. G. & Vanormelingen, P. An inordinate fondness? The number, distributions, and origins of diatom species. *J. Eukaryot. Microbiol.* **60**, 414–420 (2013).

3. Mann, D. G. The species concept in diatoms. *Phycologia* **38**, 437–495 (1999).

4. Pinseel, E. *et al. Pinnularia catenaborealis* sp. nov. (Bacillariophyceae), a unique chain-forming diatom species from James Ross Island and Vega Island (Maritime Antarctica). *Phycologia* **56**, (2017).

5. Cantrill, D. J., Ashwort, A. C. & Lewis, A. R. Megaspores of an early Miocene aquatic lycopod (Isoetales) from Antarctica. *Grana* **56**, 112–123 (2017).

6. Lewis, A. R. *et al.* Mid-Miocene cooling and the extinction of tundra in continental Antarctica. *Proc. Natl. Acad. Sci. U. S. A.* **105**, 10676–10680 (2008).

7. Lewis, A. R. & Ashworth, A. C. An early to middle Miocene record of ice-sheet and landscape evolution from the Friis Hills, Antarctica. *Geol. Soc. Am. Bull.* **128**, 719–738 (2016).

8. Souffreau, C. *et al.* A time-calibrated multi-gene phylogeny of the diatom genus *Pinnularia*. *Mol. Phylogenet. Evol.* **61**, 866–879 (2011).

9. Saint Martin, S. & Saint Martin, J. P. The diatom assemblages as environmental evolution recording of the Paratethys area during Sarmatian times. *Comptes Rendus Palevol* **4**, 191–201 (2005).

10. Nakov, T., Beaulieu, J. M. & Alverson, A. J. Accelerated diversification is related to life history and locomotion in a hyperdiverse lineage of microbial eukaryotes (Diatoms, Bacillariophyta). *New Phytol.* **219**, 462–473 (2018).

11. Huss, V. A. R. *et al.* Biochemical taxonomy and molecular phylogeny of the genus *Chlorella* sensu lato (Chlorophyta). *J. Phycol.* **35**, 587–598 (1999).

12. Guillou, L. *et al. Bolidomonas*, a new genus with two species belonging to a new algal class, the Bolidophyceae (Heterokonta). *J. Phycol.* **35**, 368–381 (1999).

13. Daugbjerg, N. & Andersen, R. A. A molecular phylogeny of the heterokont algae based on analyses of chloroplast-encoded *rbcL* sequence data. *J. Phycol.* **33**, 1031–1041 (1997).

14. Ehara, M., Inagaki, Y., Watanabe, K. I. & Ohama, T. Phylogenetic analysis of diatom *coxI* genes and implications of a fluctuating GC content on mitochondrial genetic code evolution. *Curr. Genet.* **37**, 29–33 (2000).

15. Moon-van der Staay, S. Y., van der Staay, G. W. M., Guillou, L., Vaulot, D. & Medlin, L. K. Abundance and diversity of prymnesiophytes in the picoplankton community from the equatorial Pacific Ocean inferred from 18S rDNA sequences. *Limnol. Oceanogr.* **45**, 98–109 (2000).

16. Zimmermann, J., Jahn, R. & Gemeinholzer, B. Barcoding diatoms: evaluation of the V4 subregion on the 18S rRNA gene, including new primers and protocols. *Org. Divers. Evol.* **11**, 173–192 (2011).

17. Scholin, C. A., Herzog, M., Sogin, M. & Anderson, D. M. Identification of group–specific and strain-specific genetic-markers for globally distributed *Alexandrium* (Dinophyceae). 2. Sequence analysis of a fragment of the LSU ribosomal RNA gene. *J. Phycol.* **30**, 999–1011 (1994).

18. Hamsher, S. E., Evans, K. M., Mann, D. G., Poulíčková, A. & Saunders, G. W. Barcoding diatoms: exploring alternatives to COI-5P. *Protist* **162**, 405–422 (2011).

19. Yoon, H. S., Hackett, J. D. & Bhattacharya, D. A single origin of the peridinin- and fucoxanthin-containing plastids in dinoflagellates through tertiary endosymbiosis. *PNAS* **99**, 11724–11729 (2002).

20. Ruck, E. C. & Theriot, E. C. Origin and evolution of the canal raphe system in diatoms. *Protist* **162**, 723–737 (2011).

21. Jones, H. M., Simpson, G. E., Stickle, A. J. & Mann, D. G. Life history and systematics of *Petroneis* (Bacillariophyta), with special reference to British waters. *Eur. J. Phycol.* **40**, 61–87 (2005).

22. Bruder, K. & Medlin, L. Morphological and molecular investigations of Naviculoid diatoms. II. Selected genera and families. *Diatom Res.* **23**, 283–329 (2008).

23. Bruder, K. & Medlin, L. K. Molecular assessment of phylogenetic relationships in selected species/genera in the naviculoid diatoms (Bacillariophyta). I. The genus *Placoneis*. *Nov. Hedwigia* **85**, 331–352 (2007).

24. Bouckaert, R. & Xie, D. Standard Nucleotide Substitution Models v1.0.1. http://doi.org/10.5281/zenodo.995740 (2017).
