# Supplementary material for: Global radiation in a rare biosphere soil diatom
Source: Nat Commun. 2020 May 13;11:2382. doi: 10.1038/s41467-020-16181-0 (PMC7221085; doi:10.1038/s41467-020-16181-0)
Supplement: Supplementary file 3 — Reporting Summary [file 41467_2020_16181_MOESM3_ESM.pdf]

nature research

Corresponding author(s): Eveline Pinseel, Wim Vyverman

Last updated by author(s): Apr 16, 2020

Reporting Summary

Nature Research wishes to improve the reproducibility of the work that we publish. This form provides structure for consistency and transparency in reporting. For further information on Nature Research policies, see Authors & Referees and the Editorial Policy Checklist .

Statistics

For all statistical analyses, confirm that the following items are present in the figure legend, table legend, main text, or Methods section.

n/a	Confirmed	
x		The exact sample size (*n*) for each experimental group/condition, given as a discrete number and unit of measurement
x		A statement on whether measurements were taken from distinct samples or whether the same sample was measured repeatedly
	x	The statistical test(s) used AND whether they are one- or two-sided<br>*Only common tests should be described solely by name; describe more complex techniques in the Methods section.*
x		A description of all covariates tested
x		A description of any assumptions or corrections, such as tests of normality and adjustment for multiple comparisons
	x	A full description of the statistical parameters including central tendency (e.g. means) or other basic estimates (e.g. regression coefficient) AND variation (e.g. standard deviation) or associated estimates of uncertainty (e.g. confidence intervals)
	x	For null hypothesis testing, the test statistic (e.g. *F*, *t*, *r*) with confidence intervals, effect sizes, degrees of freedom and *P* value noted<br>*Give P values as exact values whenever suitable.*
	x	For Bayesian analysis, information on the choice of priors and Markov chain Monte Carlo settings
x		For hierarchical and complex designs, identification of the appropriate level for tests and full reporting of outcomes
x		Estimates of effect sizes (e.g. Cohen's *d*, Pearson's *r*), indicating how they were calculated

Our web collection on statistics for biologists contains articles on many of the points above.

Software and code

Policy information about availability of computer code

Data collection

No software was used for data collection.

Data analysis

Software used in this study:

- Chromatograms obtained by Sanger sequencing were individually edited using BioNumerics v3.5 (Applied Maths, Kortrijk, Belgium) (available at https://www.applied-maths.com/bionumerics).
- BioEdit v7.2.5 was used to align the genes (available at https://bioedit.software.informer.com/7.2/).
- jModelTest v2.1.3 was used to estimate appropriate substitution models for the single-gene alignments of the non-coding genes (18S, 28S) (available at https://github.com/ddarriba/jmodeltest2).
- PartitionFinder v1.1.0 was used to estimate appropriate substitution models and partition schemes for the single-gene alignments of the coding genes (cox1, psbA, psbC, rbcL), and for all concatenated alignments (available at https://github.com/brettc/partitionfinder).
- TCS1.21 was used for Statistical Parsimony Network Analysis, and to detect different haplotypes in the alignments (available at http://w3.ualg.pt/~rcastil/SOFTWARE_WINDOWS/TCS1.21/docs/TCS1.21.html).
- Tracer v1.6 was used to assess convergence of MCMC runs (available at http://tree.bio.ed.ac.uk/software/tracer/).
- MrBayes v3.2.6 was used to obtain ultrametric trees for the sGMYC analyses (available at http://nbisweden.github.io/MrBayes/).
- RAxML v8.2.4 was used to run maximum likelihood phylogenetic analyses (available at https://cme.h-its.org/exelixis/web/software/raxml/index.html).
- IQ-TREE v1.6.7 was used to run maximum likelihood phylogenetic anaylses (available at http://www.iqtree.org/).
- BEAST v2.5.0 was used for obtaining phylogenetic trees by bayesian inference (available at https://www.beast2.org/).
- SSM v1.0.1 was used to use standard substitution models in the BEAST v2.5.0 analysis (available at https://github.com/BEAST2-Dev/substmodels)
- BEAST v1.10.4 was used for the time-calibrated phylogenetic analyses (available at https://github.com/beast-dev/beast-mcmc/releases/tag/v1.10.4).
- EstimateS v9.1.0 was used to calculate rarefaction curves (available at http://viceroy.eeb.uconn.edu/estimates/).

- BAMM v2.5.0 was used to run the BAMM program (available at http://bamm-project.org/)
- Mesquite v3.61 was used to perform maximum likelihood ancestral state reconstructions of habitat type (available at http://www.mesquiteproject.org/).
- PopArt v1.7 was used to calculate the haplotype network (available at http://popart.otago.ac.nz/downloads.shtml)
- Mothur v1.39.5 was used for the taxonomic classifications of the ASVs (available at https://www.mothur.org/), using the PR2 database v4.8.0 as reference (available at https://github.com/pr2database/pr2database)
- R v3.4.3, v3.4.4 and v3.6.2 were used for various analyses (see below) (available at https://www.r-project.org/)

R-packages from which functions were used for data analyses:
- SPLITS in R v3.4.3 was used to run sGMYC (available at https://rdrr.io/rforge/splits/)
- Phytools in R v3.4.3 was used to obtain lineage-through-time plots (available at https://github.com/liamrevell/phytools)
- Geiger v2.0.6.4 in R v3.6.2 was used to calculate net diversification rates (available at https://cran.r-project.org/web/packages/geiger/index.html)
- TESS v2.1.0 in R v3.4.3 was used to run the CoMET analysis (available at https://cran.r-project.org/web/views/Phylogenetics.html)
- BAMMtools v2.1.6 in R v3.4.3 was used to estimate the starting values of the priors for the BAMM analysis (available at https://cran.r-project.org/web/packages/BAMMtools/index.html)
- hisse v1.9.6 in R v3.6.2 was used to run the MiSSE model (available at https://cran.r-project.org/web/packages/hisse/index.html)
- RPANDA v1.7 in R v3.6.2 was used to run ClaDS (available at https://www.rdocumentation.org/packages/RPANDA/versions/1.7)
- BioGeoBEARS v1.1.2 in R v3.6.2 was used to run BioGeoBEARS (available at https://github.com/nmatzke/BioGeoBEARS)
- ade4 in R v3.4.3 was used to run the AMOVA analysis (available at https://cran.r-project.org/web/packages/ade4/index.html)
- DADA2 v1.6.0 in R v3.4.4 was used to obtain Amplicon Sequence Variants from the metabarcoding data (available at https://github.com/benjjneb/dada2)

Webservers used for data analysis:
- IQ-TREE webserver (http://www.iqtree.org/)
- CIPRES Science Gateway (https://www.phylo.org/)
- ABGD webserver (http://wwwabi.snv.jussieu.fr/public/abgd/)
- PTP webserver (https://species.h-its.org/ptp/)

For manuscripts utilizing custom algorithms or software that are central to the research but not yet described in published literature, software must be made available to editors/reviewers. We strongly encourage code deposition in a community repository (e.g. GitHub). See the Nature Research guidelines for submitting code & software for further information.

Data

Policy information about availability of data

All manuscripts must include a data availability statement. This statement should provide the following information, where applicable:

- Accession codes, unique identifiers, or web links for publicly available datasets
- A list of figures that have associated raw data
- A description of any restrictions on data availability

Newly determined Sanger sequences have been deposited in GenBank under accession numbers MN319619–MN319641, MN319643–MN319644, MN319651–MN319652, MN662533, MN940449–MN940569, MN940581–MN941434, MN941851–MN941898, MN943234–MN943270, MN974675–MN974732, MN974734–MN975258, MN986897, and MN992091–MN992098. All Sanger sequences, and associated environmental data, of the P. borealis complex are also available on BOLD as dataset DS-PIBOR [http://dx.doi.org/10.5883/DS-PIBOR]. The raw Illumina 18S-reads are available from the NCBI Sequence Read Archive under bioproject number PRJNA599198 [https://www.ncbi.nlm.nih.gov/sra/?term=PRJNA599198]. The alignments used for the phylogenetic analyses, the phylogenetic trees, the sequences of all ASVs recovered in this study, and the ASV-table are available from Mendeley Data under doi 10.17632/9tyhcrjrnr.1. The source data underlying Fig. 2c, and Supplementary Figs 1, 4, 6c, 9, 10a-b, 11, 13a-c are provided as a Source Data file. Previously published Sanger sequences are available under the GenBank accession codes listed in Supplementary Data 2. The previously published metabarcoding dataset used in this study is available from Figshare under doi 10.6084/m9.figshare.7845167. Other relevant data supporting the findings of the study are available in the Supplementary Information section, the Supplementary Data files, or from the corresponding authors upon request.

Field-specific reporting

Please select the one below that is the best fit for your research. If you are not sure, read the appropriate sections before making your selection.

☐ Life sciences ☐ Behavioural & social sciences ☒ Ecological, evolutionary & environmental sciences

For a reference copy of the document with all sections, see nature.com/documents/nr-reporting-summary-flat.pdf

Ecological, evolutionary & environmental sciences study design

All studies must disclose on these points even when the disclosure is negative.

Study description	This study consists of a global sampling of environmental samples containing the diatom species complex P. borealis. From these samples, diatom cultures were established. These were sequenced for various genes using Sanger sequencing. The sequences were subsequently used to build phylogenetic trees, assess diversity levels of P. borealis, and to investigate the diversification and biogeographic history of P. borealis. In addition, part of the samples was used for environmental DNA analysis using metabarcoding.

Research sample

This study partially consists of already existing datasets, and newly developed datasets.

Existing datasets included extracted DNA from previously established diatom cultures (available upon request from the Laboratory of Protistology and Aquatic Ecology at Ghent University: wim. vyverman@ugent.be), as well as previously published DNA sequences (available on GenBank). The already existing datasets include Souffreau et al. 2011 (Mol. Phylogenet. Evol. 61: 866-879), Souffreau et al. 2013 (Protist 164: 101-115), Pinseel et al. 2017 (Phycologia 56: 94-107), and Pinseel et al. 2019 (Protist 170: 121-140). The extracted DNA/GenBank sequences belonged to several diatom species of the genera Pinnularia, Sellaphora, Eolimna and Mayamaea. The majority of the data belonged to the diatom species complex Pinnularia borealis.

Together with the already existing dataset, the newly developed dataset included 867 monoclonal cultures of the P. borealis species complex from various locations worldwide, as well as 132 environmental samples which included P. borealis cells.

In this study, we were interested in the diversity and evolutionary history of the diatom species complex P. borealis. Therefore, our sample design was designed to retrieve a maximum number of different species belonging to the complex, and to simultaneously recover intraspecific diversity to aid with the automated molecular species delimitation methods. Our sampling thus represents a representative subset of the P. borealis species present on a global scale.

Sampling strategy

As we aimed to provide an inventory of the global diversity of the P. borealis species complex, our sampling strategy included a) isolating P. borealis from soils sampled at the global scale to at least retrieve an equal number of varieties and formae described within the P. borealis complex by means of morphological features (see Kociolek JP, et al. 2018. DiatomBase. Accessed at http://www.diatombase.org/ on 2018-02-28; 66 varieties and formae of P. borealis), and b) monitoring the accumulation of new molecular lineages as a function of investigated soil samples/strains. Standardized intensive screening (see 'Data collection') of soil samples ensured that presence/absence of borealis could be determined with confidence. However, the number of molecular lineages accumulated as a function of the number of investigated soil samples did not level off, despite analysis of over 1,500 soil samples. In this paper we do not claim to provide a complete view of P. borealis global diversity as the results of rarefaction analysis suggest the existence of numerous additional species, provided additional sampling. In fact, this unexpected finding is one of the novelties which makes our paper a game changing publication.

More in detail, for each sampling locality, multiple samples were collected randomly for establishing monoclonal cultures. Care was taken to include the different microhabitats that were present in the area. A various number of samples was collected per area, depending on time-availability, and the number of different microhabitats that were present. Samples for diatom cultures were collected from terrestrial mosses (above- and belowground parts), soils (top layer, ± upper 2 cm), and littoral sediments from lakes and ponds. All samples were stored in sterile falcon tubes (15 mL/50 mL) or sterile sampling bags. All samples were stored dark, and if possible, cool (< 10 °C) during transport. No chemicals were added. Upon arrival in the lab, all samples were stored at 4 °C, with exception of samples from subtropical regions (which were stored at 18 °C).

For several of the samples collected for culture establishment of P. borealis, duplicate samples were collected in the field for environmental DNA analysis. Samples for environmental DNA analysis were collected from terrestrial mosses (above- and belowground parts), soils (top layer, ± upper 2 cm), and littoral sediments from lakes and ponds, as described above. All samples were stored dark, and if possible, frozen (-20 °C). When samples could not be frozen, Sucrose Lysis Buffer (SLB; 20 mM EDTA, 200 mM NaCl, 0.75M sucrose, and 50 mM Tris-HCl at pH 9) was added to prevent biological activity, and to preserve DNA quality. Upon arrival in the lab, all samples were frozen at -80 °C, prior to DNA extraction.

Sample collection was performed by several of the authors: E.P., E.V., P.V., T.J.K., E.M.B., B.V.d.V. and W.V., as well as several people listed in the acknowledgements section of the paper.

Data collection

Small quantities of the natural material (subsamples) were incubated for several weeks to months in WC medium, without pH adjustment or vitamin addition, at 4 °C (for polar and temperate regions) or 18 °C (for subtropical regions), 5 – 10 µmol photons m-2 s-1 and a 12:12h (light:dark) cycle. Although the abundances of P. borealis cells were low in the overall majority of the samples, this was accommodated by careful sample treatment by the first author (E.P.). All environmental samples were subsampled in multiple wells of 12-well plates. In doing so, care was taken to take material from different parts of the sample, and if the sample was heterogeneous (for example, a mix of soil and moss), multiple subsamples from these different parts were taken. These samples were subsequently screened repeatedly in a light microscope over a course of several weeks. In case only dead valves of P. borealis were observed, samples were screened over longer time periods (up to four to six months). Although time-consuming, this approach ensured that the chances of observing living P. borealis cells were maximized.

Isolations of P. borealis cells were performed whenever it became possible to find living cells. Monoclonal cultures were established by isolating single cells under an Olympus SZX9 stereomicroscope using a needle and a micropipette. Cultures were grown in WC medium at standard culture conditions of 18 °C, 5-10 µmol photons m-2 s-1 and a 12:12h (light:dark) cycle, and reinoculated when reaching late exponential phase. When sufficient biomass was obtained, subsamples for morphological and molecular analysis were taken.

All culture work was performed by E.P., under initial guidance of P.V. Molecular data analysis was performed by E.P. and several lab technicians (listed in acknowledgment).

Timing and spatial scale

The spatial scale of this study is global: samples were collected from all continents.

Samples were collected over a seven-year time period (between 2011 and 2017). The samples were collected during the growing season (spring – summer – autumn). The samples were collected once for each locality (no repeated sampling was done).

In this study, we aimed to provide a first overview of the global diversity of the diatom species complex surrounding P. borealis. To this end, the geographic coverage had to be maximized, and as a consequence repeated sampling was not feasible. Sampling was

spread over multiple years in order to allow for the collection and management of a large number of samples from around the world.

Data exclusions	No data were excluded from the analyses.
Reproducibility	N/A: no experiments were performed.
Randomization	N/A
Blinding	N/A

Did the study involve field work? ☒ Yes ☐ No

Field work, collection and transport

Field conditions	Samples were collected in various field conditions (rain, sunshine) and various temperatures ranging between ~0 °C and ~30°C For this study, the conditions during field sampling are not relevant for the results.
Location	Samples were collected at various locations. A detailed overview of the sampling locations can be found in the supplementary data files associated with this article (Supplementary Data 1 and Supplementary Data 3).
Access and import/export	All samples were obtained in compliance with local, national and international laws and regulations. When necessary, permits were obtained: Terres Australes et Antarctiques Françaises (13 September 2016): arrête n° 2016-96 ; Terres Australes et Antarctiques Françaises (15 December 2016): arrête n° 2016-162 ; Terres Australes et Antarctiques Françaises (15 December 2016): arrête n° 2016-166 ; Terres Australes et Antarctiques Françaises (15 December 2016): arrête n° 2016-165 ; Terres Australes et Antarctiques (22 June 2015): arrête n° 2015-56; République Française (25 June 2018): TREL1734890A/44 ; Wellington Park Management Trust (16 January 2017): 07-84-46 ; Department of Primary Industries, Parks, Water and Environment Tasmania (25 October 2016): FL 16259 ; Department of Primary Industries, Parks, Water and Environment Tasmania (16 January 2017): FL 17008 ; Department of Primary Industries, Parks, Water and Environment Tasmania (01 December 2016): TFL 16331; Australian Government – Department of the Environment and Energy (03 February 2017): PWS2017-AU-000202 ; Australian Government – Department of the Environment and Energy (13 June 2017): PWS2017-AU-001086 ; Naalakkersuisut Government of Greenland (27 March 2015): G15-005; Naalakkersuisut Government of Greenland (14 April 2015): G15-012 ; Naalakkersuisut Government of Greenland (06 August 2015): G15-037 ; Naalakkersuisut Government of Greenland (16 June 2017): G17-027 ; Naalakkersuisut Government of Greenland (02 November 2017): G17-049 ; Aurora Research Institute (2016): permit number 14965 ; Republic of South Africa – Department of Environmental Affairs (15 November 2016): AC003/2016 ; South Georgia and the South Sandwich Islands (20 January 2017): 2016/033 ; United Kingdom of Great Britain and Northern Ireland (04 November 2014): S9-29/2014 ; Governor of Svalbard (20 May 2015): 2011/00032-4 ; Governor of Svalbard (28 April 2016): 16/00468-1 ; Norwegian Polar Institute (03 October 2016): 2016/115-26 ; Ministry of Environment of The Czech Republic (15 December 2011): PERMIT on the on the basis of an application of November 6, 2011 ; Ministry of the Environment of The Czech Republic (14 December 2012): PERMIT on the basis of an application of December 1, 2011 ; Ministry of the Environment of The Czech Republic (15 November 2013): PERMIT on the basis of an application on November 11, 2013 ; Ministry of Environment of The Czech Republic (10 December 2014): PERMIT on the basis of an application of November 25, 2014 ; Belgium - Federal Public Service: health, food chain safety and environment (October 2014): Nr 2014/03 ; Federal Service for Hydrometeorology and Environmental Monitoring to the Arctic and Antarctic Research Institute (St. Petersburg) (23 January 2016): No R/2016/014.
Disturbance	The amount of material collected per sample was small (~5 - 50 grams weight-wet). As a consequence, disturbance was kept to an absolute minimum.

Reporting for specific materials, systems and methods

We require information from authors about some types of materials, experimental systems and methods used in many studies. Here, indicate whether each material, system or method listed is relevant to your study. If you are not sure if a list item applies to your research, read the appropriate section before selecting a response.

Materials & experimental systems

n/a	Involved in the study
☒	☐ Antibodies
☒	☐ Eukaryotic cell lines
☒	☐ Palaeontology
☒	☐ Animals and other organisms
☒	☐ Human research participants
☒	☐ Clinical data

Methods

n/a	Involved in the study
☒	☐ ChIP-seq
☒	☐ Flow cytometry
☒	☐ MRI-based neuroimaging
