# Supplementary material for: Global radiation in a rare biosphere soil diatom
Source: Nat Commun. 2020 May 13;11:2382. doi: 10.1038/s41467-020-16181-0 (PMC7221085; doi:10.1038/s41467-020-16181-0)
Supplement: Supplementary file 4 — Description of Additional Supplementary Files [file 41467_2020_16181_MOESM4_ESM.pdf]

Description of Additional Supplementary Files

File Name: Supplementary Data 1
Description: Overview of the environmental samples from which P. borealis strains were obtained. Sample origin, GPS coordinates, substratum, general habitat type and the number of established strains per sample are given. Samples indicated with an asterisk (*) were not collected in the framework of this study, but originate from earlier work (see Supplementary Data 2 for references). N.A. = not obtained.

File Name: Supplementary Data 2
Description: Overview of all strains of the P. borealis species complex examined in this study. Strains indicated in bold represent reference strains used in the phylogenetic analysis. The 28S- and cox1-lineages to which each strain belongs are indicated by the reference strain for each lineage. BOLD and GenBank accession numbers are given for each record. If a strain/sequence was not obtained in this study, the reference towards the original study is indicated. N.A. = not obtained/not included.

File Name: Supplementary Data 3
Description: Overview of the environmental samples which were used for 18S environmental metabarcoding. Sample origin, GPS coordinates, substratum and general habitat type are given. Pinnularia borealis cultures were established from all samples indicated with an asterisk (*). Additional information can be found with the associated sequence data on the NCBI Sequence Read Archive under bioproject number PRJNA599198.
